# Possible Implication of Bacterial Infection in Acute Graft-Versus-Host Disease after Allogeneic Hematopoietic Stem Cell Transplantation

**DOI:** 10.3389/fonc.2014.00089

**Published:** 2014-04-24

**Authors:** Shigeo Fuji, Markus Kapp, Hermann Einsele

**Affiliations:** ^1^Division of Hematology, Department of Internal Medicine II, University Hospital of Würzburg, Würzburg, Germany; ^2^Division of Hematopoietic Stem Cell Transplantation, National Cancer Center Hospital, Tokyo, Japan

**Keywords:** bacterial infection, GVHD, allogeneic hematopoietic stem cell transplantation, pathogen-associated molecular patterns, LPS

## Abstract

Graft-versus-host disease (GVHD) is still one of the major causes of morbidity and mortality in allogeneic hematopoietic stem cell transplantation (HSCT). In the pathogenesis of acute GVHD, it has been established that donor-derived T-cells activated in the recipient play a major role in GVHD in initiation and maintenance within an inflammatory cascade. To reduce the risk of GVHD, intensification of GVHD prophylaxis like T-cell depletion is effective, but it inevitably increases the risk of infectious diseases and abrogates beneficial graft-versus-leukemia effects. Although various cytokines are considered to play an important role in the pathogenesis of GVHD, GVHD initiation is such a complex process that cannot be prevented by means of single inflammatory cytokine inhibition. Thus, efficient methods to control the whole inflammatory milieu both on cellular and humoral view are needed. In this context, infectious diseases can theoretically contribute to an elevation of inflammatory cytokines after allogeneic HSCT and activation of various subtypes of immune effector cells, which might in summary lead to an aggravation of acute GVHD. The appropriate treatments or prophylaxis of bacterial infection during the early phase after allogeneic HSCT might be beneficial to reduce not only infectious-related but also GVHD-related mortality. Here, we aim to review the literature addressing the interactions of bacterial infections and GVHD after allogeneic HSCT.

## Introduction

Graft-versus-host disease (GVHD) is still one of the major causes of morbidity and mortality responsible for 10–20% of all deaths in allogeneic hematopoietic stem cell transplantation (HSCT) (1, 2). In the pathogenesis of acute GVHD, it has been established that donor-derived T-cells activated in the recipient play a major role in GVHD initiation and maintenance within a complex inflammatory cascade (3). To reduce the risk of GVHD, intensification of GVHD prophylaxis such as profound T-cell depletion is effective, but it inevitably increases the risk of infectious diseases and abrogates beneficial graft-versus-leukemia (GVL) effects. Another potentially beneficial intervention to reduce the risk of GVHD could be the suppression of inflammatory cytokines, which promotes the initiation and maintenance of GVHD-associated T-cell activations. Even though it is well established that cytokines play an important role in the pathogenesis of GVHD, GVHD is a complex process that cannot be prevented with a single inflammatory cytokine inhibition as demonstrated previously (4). Thus, efficient methods to control the whole inflammatory milieu are needed.

Infectious diseases can theoretically contribute to an elevation of inflammatory cytokines after allogeneic HSCT (5–7). Possible interaction between viral infections and graft rejections of transplanted organ or GVHD are thought to be mediated by the alloreactivity of virus-specific T-cells (8, 9). Bacterial infection can also induce GVHD rather non-specifically, considering the induction of systemic proinflammatory cytokines (10). The appropriate treatment or prophylaxis of bacterial infection during the early phase after allogeneic HSCT might be beneficial to reduce not only infection-related but also GVHD-related mortality. In terms of fungal infection, we could assume that the fungal infection is also implicated in the pathogenesis of acute GHVD, and recent reports suggested the implication of fungal infection in the pathogenesis of acute GVHD (11).

Here, we aim to review the literature addressing the interactions of bacterial infections and GVHD after allogeneic HSCT and discuss the potential benefits/disadvantages of published therapeutic options.

## Interaction between Bacterial Products and GVHD in Mouse Models

The fundamental work of van Bekkum and colleagues impressively demonstrated that activations of innate immunity by the gastrointestinal microflora are crucial and initiating steps in the induction of alloreactions. In animal models, mice grown under germ-free conditions and receiving bone marrow as a source of hematopoietic stem cells following total body irradiation did not develop acute GVHD (12). However, when a high number of T-cells were added, germ-free condition alone did not prevent but still delayed the onset of acute GVHD.

**Figure 1:**
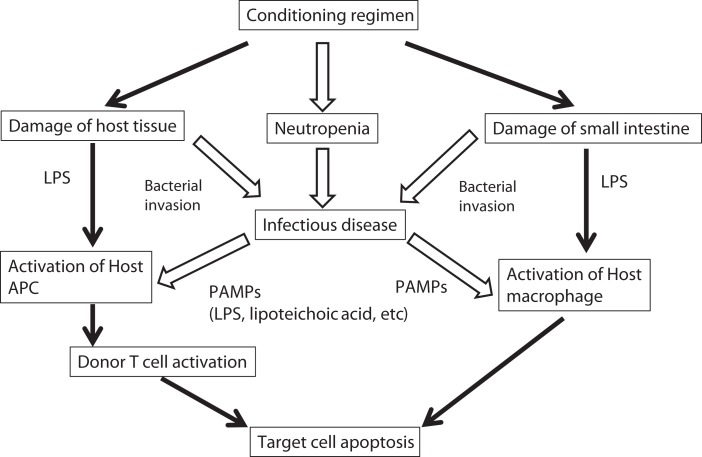
**Possible implication of bacterial infection in the pathogenesis of GVHD**. Progression of acute GVHD can be summarized in three steps following the damage of host tissue by conditioning regimen: (1) activation of APCs; (2) donor T-cell activation, proliferation, differentiation, and migration; and (3) target tissue destruction. Injury to the gastrointestinal tract from conditioning causes systemic translocation of additional inflammatory stimuli, such as microbial products including LPS or other PAMPs, which further enhance activation of host APCs. In addition, conditioning regimen causes severe neutropenia that increased the risk of infectious disease. Infectious diseases increase the secretion of PAMPs, which activate host APCs. LPS, lipopolysaccharide; APC, antigen-presenting cell; PAMP, pathogen-associated molecular patterns.

The role of bacterial products and the innate immune response in the pathophysiology of acute GVHD was nicely reviewed a decade ago (13). After tissue damage induced by the conditioning regimen, bacterial products contribute to an activation and expansion of donor-derived T-cells via antigen-presenting cells (APCs) (14). Such bacterial products are called as pathogen-associated molecular patterns (PAMPs) including lipopolysaccharide (LPS). In the activation of immune cells through a complex signaling cascade, toll-like receptors (TLRs) play an important role in recognizing PAMPs including LPS (15). The combination of TLR and their ligand is summarized in Table 1. In addition to TLRs, various nucleotide-binding and oligomerization domain (NOD)-like receptors (NLRs) play a vital role in innate immunity (16). Severe injury to tissues results in increased release of endotoxin and exacerbation of the inflammation. Bacterial infection itself is expected to stimulate the innate immunity similar to the tissue damage by the conditioning regimen (Figure 1). The stimulated APCs including dendritic cells and monocytes provoke an enhanced adaptive immunity by stimulating T-cells via antigen-specific signaling (7, 15). Cooke and colleagues (17) reported that the sensitivity to LPS affected the severity of GVHD and idiopathic pneumonia syndrome in mice. They used two mouse strains which differ in their sensitivity to LPS and found that LPS-resistant recipients which had a genetic mutation in the TLR4 gene had significantly less lung injury and GVHD. These effects were associated with the reduction of TNF-α secretion (17, 18). They chose a direct approach to inhibit acute GVHD by using a direct competitive antagonist of endotoxin and by attenuation of the inflammatory response following transplantation, which improved GVHD score and survival (19). Mice were treated in the first 6 days after HSCT, when the donor T-cells are considered to be maximally stimulated by the host injury, and observed a concomitant reduction in inflammatory cytokine levels. This treatment was not associated with a parallel suppression of the beneficial GVL effects, unlike non-specific immune suppression in this model. These experimental studies demonstrated the implication of LPS/TLR4 signaling in alloreaction.

**Table 1:** **Combination of toll-like receptor and its ligand**.

TLR	Ligands	Recognized pathogens
TLR1	Triacyl lipopeptides	Bacteria
TLR2	Lipoproteins, peptideglycan, LTA, β-d-glucan, and mannan	Bacteria and fungus
TLR3	dsRNA	Virus
TLR4	LPS, RSV fusion protein, and mannans	GNR and virus
TLR5	Flagellin	Bacteria
TLR6	Diacyl lipopeptides, LTA, and β-d-glucan	Bacteria and fungus
TLR7	ssRNA	Virus, fungus, and bacteria
TLR8	ssRNA	Virus
TLR9	DNA and hemozoin	Bacteria, fungus, virus, and protozoan parasites
TLR10	Bacterial lipopeptide?	Bacteria? Virus?

*LTA, lipoteichoic acid; LPS, lipopolysaccharide; RSV, respiratory syncytial virus; GNR, Gram-negative rod*.

As another signaling pathway, several studies using a mouse model demonstrated the importance of CpG-containing DNA/TLR9 signaling. Calcaterra et al. (20) reported that survival and clinical score of acute GVHD in TLR9 knockout recipient mice were improved as compared with the wild-type recipient mice. Taylor et al. (21) also reported that the administration of CpG-oligodeoxynucleotide, which mimic viral and bacterial DNA and are recognized by TLR9 markedly aggravated GVHD. In summary, these mouse models demonstrated the importance of bacterial products in the pathogenesis of acute GVHD.

## Gut Bacteria and GVHD

It was reported that low bacterial environments strikingly limit the risk of GVHD in patients undergoing allogeneic HSCT (22). Beelen et al. assessed the influence of intestinal bacterial decontamination on the occurrence of grades II–IV acute GVHD retrospectively in 194 patients following HLA identical sibling marrow transplantation under conditions of strict protective isolation and intestinal antimicrobial decontamination (23). Using the duration of anaerobic growth suppression as a time-dependent explanatory variable, anaerobic decontamination was a significant independent predictor for grade II–IV acute GVHD (HR 1.7, 95%CI 1.2–2.5, *P* < 0.05). Following the promising finding, which suggested a beneficial effect of gut decontamination, a single-center open-label prospective study was conducted. A total of 134 marrow transplant recipients with hematologic malignancies were randomly assigned to a bacterial decontamination using metronidazole and ciprofloxacin (*n* = 68) or ciprofloxacin alone (*n* = 66) (24). Treatment was initiated on day 14 and was maintained until day 35 posttransplant. According to an intention-to-treat principle, 17 patients (25%) randomized to the combined decontamination medication and 33 patients (50%) randomized to ciprofloxacin alone developed grades II–IV GVHD (*P* < 0.05). The higher frequency of grades II–IV acute GVHD in patients randomized to ciprofloxacin alone resulted from a more than twofold increased number of patients developing liver or intestinal involvement with acute GVHD compared with patients randomized to the combined decontamination medication (*P* < 0.05). Regarding the addition of metronidazole, it might reduce the risk of *Clostridium difficile* infection (CDI), which has been reported to be associated with subsequent GVHD onset (25, 26). Alonso and colleagues reported that patients who developed CDI were more likely to develop GI GVHD compared with those who never developed CDI (*P* < 0.001). In this study, the diagnosis of CDI preceded the diagnosis of GI GVHD in the majority of patients (12 of 14 patients, 85.7%). Among the 12 patients who developed GI GVHD following CDI, GI GVHD diagnosis occurred at a median of 21.5 days after CDI. Thus, gut decontamination might reduce the risk of acute GVHD. However, due to concerns about resistance or disturbance of the microbiota by prophylactic antibiotics, the type of antibiotics and the duration of prophylaxis have to be carefully discussed (27).

Correlation between the intestinal microbiome and autoimmune diseases has been demonstrated recently (28, 29). Even though there is not much data about the correlation between the intestinal microbiome and GVHD, it has been established that the microbiome has an influence on the status of immune cells (30). A recent report showed that the abundance of bacteria belonging to the genus *Blautia*, a commensal commonly found in the intestinal tract of humans, predicted for protection from severe GVHD in recipients of allogeneic HSCT (31). Furthermore, in murine models, introducing a species of *Blautia* of murine origin reduced GVHD severity. Intriguingly, loss of *Blautia* correlates strongly with reductions in oral nutritional intake in both humans and mice. Another group reported that loss of bacterial diversity was associated with use of systemic antibiotics and it was pronounced in patients with gastrointestinal GVHD (32). In addition, *Candida* colonization might be also important in the pathogenesis of GVHD (11). As a possible intervention to reduce the risk of GVHD, gut flora manipulation and nutritional intervention strategies might be promising. Previous reports showed that gut flora manipulation by *Lactobacillales* may reduce intestinal inflammation and improve outcomes for allogeneic HSCT recipients in a murine model (33, 34). It is worthy to test whether the manipulation of intestinal microbiome is able to reduce the risk of acute GVHD. Considering the considerable difference of conditioning regimen or GVHD prophylaxis in the regimen-related toxicity of oral mucosa and gastrointestinal tract, importance of gut bacterial manipulation might be more important in patients with a myeloablative conditioning regimen than those with a reduced-intensity conditioning regimen, or in patients with short-term methotrexate than those with mycophenolate mofetil (35–40).

## Bacterial Infection and GVHD

Intensive chemotherapy and irradiation result in damage to the gastrointestinal tract, allowing bacteria to enter the systemic circulation. This results in stimulation of the host immune response by the production of inflammatory cytokines. Blood stream infection (BSI) is expected to promote the inflammatory conditions, which leads to an aggravation of acute GVHD. Statistically, it is difficult to assess the association between BSI and acute GVHD because acute GVHD itself is a risk factor of subsequent BSI. Poutsiaka and colleagues (41) found that early BSI was associated with an increased risk of grade II–IV acute GVHD.

As a marker of infectious disease, C-reactive protein (CRP) is routinely used in Europe and Japan (42–44). CRP is an acute phase reactant that is elevated in patients with infectious disease along with IL-6, the main cytokine that induces CRP release (45, 46). Even though CRP has a limited value for the differential diagnosis of bacterial infections due to the non-specific elevation in patients with inflammation from other causes, profound elevation of CRP early after HSCT was in general caused by infectious diseases (47–52). Considering the role of inflammation in the pathogenesis of GVHD, it is intriguing whether systemic inflammation caused by infection also exaggerates acute GVHD. Several retrospective studies assessed the association between the elevation of CRP and transplant complications including acute GVHD (50, 53–57). Our group reported that a significant elevation of CRP during the neutropenic period was associated with a subsequent incidence of acute GVHD (50). Min et al. also reported that patients with GVHD had a significantly higher CRP level early after allogeneic HSCT compared to those without GVHD (56). Furthermore, Michigan group assessed plenty of biomarkers to establish a biomarker panel for the prediction of acute GVHD (58). Eight proteins (IL-2Rα, CRP, IL-8, ICAM-1, TIMP-1, TNFR1, HGF, and CA19.9) resulted in highly significant results (*P*-value <0.01 for two-sample *t* tests comparing patients with and without GVHD). Among these eight biomarkers, CRP had the highest fold difference (×5.44) and the second lowest *P*-value (5.9 × 10^−6^) comparing patients with acute GVHD to those without acute GVHD, even though they excluded CRP probably considering that CRP is elevated in patients with infectious diseases. However, these results also indicate that the elevation of CRP preceded the incidence of acute GVHD. Taking the results into account that elevated CRP caused by infectious diseases precedes the occurrence of acute GVHD, strategies to improve prevention of infectious diseases early after allogeneic HSCT should be explored to reduce the risk of subsequent acute GVHD and subsequently necessary immunosuppressive therapy. Studies examining an intensified prophylaxis for bacterial infection early after allogeneic HSCT using intravenous antibiotics such as tazobactam/piperacillin (59), meropenem (60), vancomycin (61, 62), or teicoplanin (63) unfortunately did not report the incidence of acute GVHD, which would be important to evaluate different antibacterial strategies and their impact in GVHD incidence and severity. Although the information of bacteria commonly found on the surfaces of the human body might help us to choose the antibiotics (64) (Table 2), we need more information about bacteria commonly found after allogeneic HSCT (27, 32). Another possible intervention could be intensive glucose control and glutamine, which were reported to ameliorate the elevation of CRP level (65–67).

**Table 2:** **Bacteria commonly found on the surfaces of the human body (64)**.

Oral cavity	Gut	Skin
*Streptococcus*	*Bacteroides*	*Propionibacterineae*
*Veillonella*	*Lachnospiraceae*	*Staphylococcus*
*Prevotella*	*Prevotella*	*Corynebacterineae*
*Pasteurellaceae*	*Faecalibacterium*	*Streptophyta*
*Neisseria*	*Lachnospiraceae*	*Micrococcineae*
*Fusobacterium*	*Ruminococcaceae*	*Streptococcus*
*Micrococcineae*	*Clostridiales*	*Finegoldia*
*Pasteurellaceae*	*Parabacteroides*	*Lactobacillus*
*Actinomycineae*	*Alistipes*	*Anaerococcus*
*Porphyromonas*	*Proteobacteria*	*Enhydrobacter*

## Genetic Polymorphism in Genes Relating to Host–Microbe Recognition and GVHD

Recent genome-wide association studies (GWAS) have identified a large number of major loci, which are associated with various autoimmune diseases including Crohn’s disease (CD), systemic lupus erythematosis (SLE), and others (16, 68, 69). In terms of CD, polymorphisms in NOD2 were reported to be associated with an increased risk of CD (70–72). In terms of SLE, Graham and colleagues (73) reported that interferon regulatory factor 5 (IRF5) has been associated with SLE. IRF5 is downstream of pattern-recognition receptor (PRR) signaling and induces numerous cytokines.

Similar to the findings in the field of autoimmunity, several polymorphisms in PRRs were associated with posttransplant complications in HSCT (74). The incidence of severe GVHD in pairs with either donor or recipient NOD2 mutations was significantly higher compared to that in donor/recipient pairs without any NOD2 variant (75, 76).

Hildebrandt and colleagues (77) reported the association between polymorphisms in NOD2 and the risk of bronchiolitis obliterans, a severe form of chronic GVHD.

In addition, several papers reported the association between the polymorphisms in PRRs and other infections such as fungal and viral infection (78). Polymorphism in dectin-1, a C-type lectin receptor recognizing the β-1,3-glucan motif of *Candida* was associated with increased *Candida* colonization of HSCT recipients, rendering them at high risk for candidemia (11, 79). Polymorphism in dectin-1 was also reported to be associated with an increased risk of *Aspergillus* infection (80). In addition, *Candida* colonization was reported to be associated with an increased incidence of acute GVHD (11). In terms of viral infection, the report is limited up to now, but Jaskula and colleagues (78) reported that polymorphism of NOD2 was associated with an increased risk of herpes virus reactivation. Considering the implication of PRRs in the interplay of host cells with invading viruses (81, 82), more studies which assess the association between polymorphisms of PRRs and the incidence of viral infection and subsequent GVHD are warranted.

## Potential Intervention to Reduce the Risk of GVHD

There are several possible interventions to reduce the risk of GVHD, focusing on the control of pathways activated by bacterial infections. Several strategies such as manipulation of gut bacteria and nutritional support have been already discussed above.

One strategy is to target the pathway of TLRs and other molecules in innate immunity. There are several drugs targeting TLRs under development, for example TLR4 antagonists (Eritoran-E5564, TAK-242) and TLR2 antagonistic antibody (83). Although studies conducted in patients with sepsis using TLR4 antagonists did not demonstrate the efficacy of these drugs, they might be useful in the amelioration of GVHD. Intervention to reduce the level of damage-associated molecular patterns (DAMPs), known as alarmins might be also effective considering the common downstream pathway between PAMPs and DAMPs. DAMPs are endogenous components commonly released by injured or stressed cells, such as nucleic acids, uric acid (UA), HMGB-1, heparan sulfate (HS), etc. (84–86). Regarding UA, one study showed that urate oxidase can be safely administered during myeloablative conditioning and may reduce the incidence of acute GVHD (87). Regarding HS, treatment with the serine protease inhibitor a1-antitrypsin decreased serum levels of HS, leading to a reduction in GVHD severity in mice (88).

Another strategy might be preemptive therapy using ATG, intra-arterial corticosteroids, and others. ATG has been tested in a prospective randomized study of preemptive GVHD treatment with ATG in 170 patients at high risk of GVHD (89–91). This study showed that a preemptive ATG significantly reduced the incidence of GVHD after an alternative donor HSCT. In terms of intra-arterial corticosteroids, small studies demonstrated the promising effectiveness in patients with severe gut GVHD (92, 93). Such preemptive strategy can be applied if we develop a scoring system incorporating the status of immunity activated by PAMPs.

## Discussion

As described above, various experimental and clinical data strongly suggest the implications of bacterial, fungal, and viral infection and acute GVHD. One possible intervention might be the manipulation of intestinal microbiota. Various strategies can be used for this purpose, such as intensification of gut bacterial decontamination or administration of *Lactobacillales*. Gut bacterial decontamination is practically simple but the duration of decontamination should be as short as possible, considering the risk of emergence of resistant bacteria and the cost factors. Administration of *Lactobacillales* has not yet been proved to be safe in immunocompromised recipients after allogeneic HSCT.

In conclusion, various evidences from experimental models and clinical studies suggest the implication of bacterial infection in the pathogenesis of acute GVHD. To ameliorate the inflammation caused by bacterial infection, specific antibacterial strategies, treatment targeting the pathway of innate immunity, or nutritional interventions might help to reduce the risk of acute GVHD, which should be prospectively assessed in clinical trials.

## Conflict of Interest Statement

The authors declare that the research was conducted in the absence of any commercial or financial relationships that could be construed as a potential conflict of interest.
